# Physiological Responses Associated with Nordic-walking training in Systolic Hypertensive Postmenopausal Women

**DOI:** 10.2478/hukin-2014-0104

**Published:** 2014-11-12

**Authors:** Ewelina Latosik, Igor Z. Zubrzycki, Zbigniew Ossowski, Olgierd Bojke, Anna Clarke, Magdalena Wiacek, Bartosz Trabka

**Affiliations:** 1 The Jerzy Kukuczka Academy of Physical Education in Katowice, ul. Mikołowska 72A, 40-065 Katowice, Poland.; 2 School of Health and Applied Sciences, Private Bag 13388, Windhoek, Namibia.; 3 Jędrzej Śniadecki Academy of Physical Education and Sport ul. Kazimierza Górskiego Gdańsk, Poland.; 4 Department of Microbiology and Biochemistry, University of Fort Hare, South Africa. .

**Keywords:** postmenopause, hypertension, exercise, nordic-walking, functional fitness

## Abstract

Loss of physical strength and hypertension are among the most pronounced detrimental factors accompanying aging. The aim of this study was to evaluate the influence of a supervised 8-week Nordic-walking training program on systolic blood pressure in systolic-hypertensive postmenopausal women. This study was a randomized control trial on a sample of 24 subjects who did not take any hypertension medications. There was a statistically significant decrease in systolic blood pressure and an increase in lower and upper-body strength in the group following Nordic-walking training. There was a decrease in serum levels of total cholesterol, triglycerides, and low-density cholesterol. The obtained results indicate that an 8-week Nordic-walking program may be efficiently employed for counteracting systolic hypertension through a direct abatement of systolic blood pressure and an increase of maximal aerobic capacity.

## Introduction

Among many detrimental factors accompanying aging, loss of strength (McNeil et al., 2007) and deterioration of postural balance leading to an increased risk of fall accidents (Thornby, 1995) play the most profound role. Hence, maintaining an appropriate level of neuromuscular fitness – through physical exercises (Ferrari, 2007) – may prevent a social withdrawal caused by an age-dependent decline in physical activity (Wiacek et al., 2009).

This observation spurred the studies on prevention of age-related loss of strength (Perkins and Kaiser, 1961) as well as promotion of physical activity among older adults (Satariano and McAuley, 2003). Recent studies also suggest that the correlation between lower body strength and fall risk is nation dependent (Lipsitz et al., 1994), and that the number of falling accidents within a given community can be an indicator of functional abilities of that population (Li et al., 2003).

An extended span of a human life is also accompanied by an age-dependent deterioration of health-related quality of life (HRQoL), driven, among others, by an increase in blood pressure (Landahl et al., 1986), which may lead to a range of cardiovascular diseases (Franklin, 1999) that result in an increased cost of medical welfare.

The aforementioned phenomena, i.e., increased blood pressure and a decreased level of physical fitness, incited a search for means, other than pharmaceutical, allowing to counteract age-dependent deterioration of HRQoL.

During the last decade, a derivative of walking (W) called Nordic-walking (NW) has become a very popular mode of physical activity in Central and Northern Europe. Its clinical viability, among young and elderly women, has already been confirmed in several studies (Figard-Fabre et al., 2011; Mikalacki et al., 2011).

Because of growing popularity of NW among Europeans, we made an attempt to test its efficacy in preventing hypertension in postmenopausal systolic-hypertensive women. For the purpose of our research we designed a moderate 8-week NW training program that comprised of three mesocycles, and carried out a randomized control trial under the null hypothesis that NW may be utilized as means of reducing systolic blood pressure in postmenopausal women.

## Material and Methods

### Participants

The following inclusion criteria were employed: last period >12 months ago, systolic blood pressure (SBP) ≥ 140 mmHg and diastolic blood pressure (DBP) < 90 mmHg.

Exclusion criteria were: (a) SBP ≥ 180 mmHg and/or DBP ≥ 90 (b) taking anti-hypertensive drugs, (c) oophorectomy, (d) chemotherapy within six months before screening, (e) coronary artery disease, (f) renal failure, (g) rheumatoid arthritis, (h) pulmonary disease, (i) diabetes mellitus or type II diabetes treated with insulin, (j) myocardial infarction or surgery within six months before screening, (k) smoking of more than two cigarettes per day or consuming more than the equivalent of one glass of wine per day, and (l) inability to obtain approval for participation in the study form a primary-care physician.

**Figure 1:**
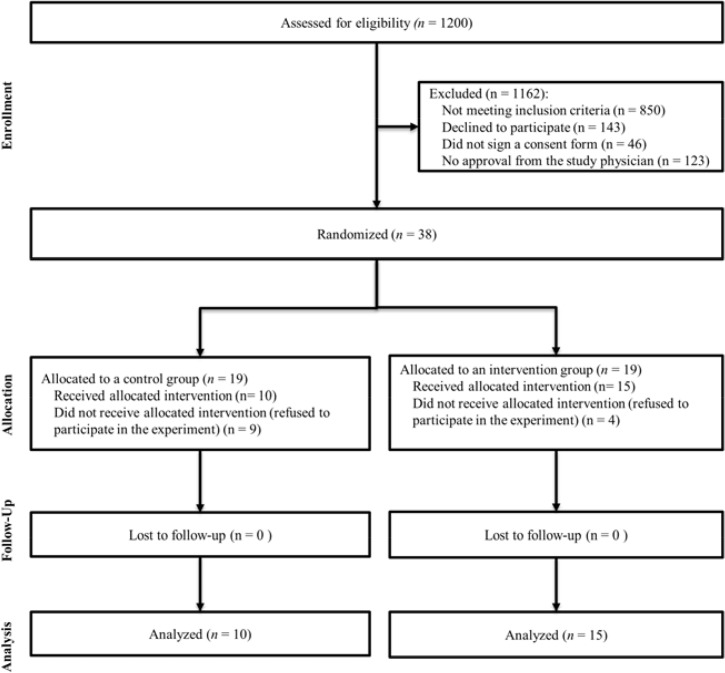
A flow diagram of the progress through the phases of a parallel randomized trial of two groups

Of 1200 women who agreed for the primary screening only 25 met all the criteria to participate in the study (Figure 1).

### Measures

#### Laboratory tests and anthropometry

Body mass and height were measured using a standard scale and a stadiometer with accuracy of 0.1 kg and 0.5 cm, respectively.

Resting SBP and DBP were registered using an Omron HEM-907XL apparatus (Omron Healthcare, Inc., IL, USA).

A fasting blood draw was completed to measure blood glucose, total cholesterol, triglycerides, and high- and low-density lipoprotein cholesterol. Serum lipid levels were measured on the first and the last days of the training program on an empty stomach using ARCHITECT ci8200 Integrated System, Abbott Diagnostic.

Subjects were advised to be well hydrated and to limit their physical activity one day before the laboratory test, as well as to remain physically inactive the evening before and the morning of the test.

#### Peak oxygen uptake

VO_2max_ peak was assessed by open-circuit spirometry using the following protocol: (a) the test was preceded by a 2 min warm up: walking at the speed of 2–3 km/h, (b) the subject started walking, without holding onto the handrails during the test, at 4 km/h and 0% grade. The elevation was increased by 2% each 2 min stage until volitional fatigue. The test was continued until the subject could no longer continue due to an excessive elevation, achieved a respiratory-exchange ratio > 1.0, a maximal heart rate greater than HR_max_ = 206 − 0.88*age, or other clinical criteria for test termination were observed.

VO_2max_ was measured using a breath-by-breath gas-exchange system (MetaMax 3B, Cortex-Medical, Germany). In this study VO_2max_ data are expressed as VO_2peak_.

#### Assessment of the level of physical fitness

Functional fitness was assessed by means of a tailored Fullerton battery test including: a) chair stand (CST), b) arm curl (CURL). All tests were performed one hour after breakfast to avoid results skewing by daily activities.

#### Statistical Analyses

Changes induced by the training/sedentary period in an analyzed parameter were examined using the Wilcoxon signed-rank test. Differences between the groups were evaluated by means of the Wilcoxon rank sum test using the p-value of 0.05 as the statistical threshold.

Relative changes induced by the experiment were measured using a “natural” relative difference, employing a natural logarithm, denoted as log percent (L%) (Tornqvist et al., 1985).

### Procedures

#### Training Program

All exercise sessions were performed outdoors on a flat area, and consisted of a 10 to 12 min warm-up followed by 45 min of NW and a 10 min stretching. The NW program was divided into three mesocycles: I: 2 weeks of walking the distance of 3 km, an average heart rate between 40–60% of an age-dependent maximal heart rate; HR_max_ = 206 − 0.88•(age) (Gulati et al., 2010), [(HR_max_ − HR_rest_)•(0.4 to 0.6)] + HR_rest_; II: 3 weeks walking the distance of 3.1 km with an average heart rate between 45–68%, and III: 3 weeks of walking the distance of 3.8 km with an average heart rate between 38–69%. The heart rate was monitored using a GARMIN Forerunner 405 apparatus.

### Diet

The following dietary guidelines were followed during the experiment: (a) consume *en gros *∼ 2.5 l water/day, (b) 130 g/day of carbohydrates, (c) 0.8 g of protein per kilogram of body mass assessed at baseline, (d) 20–30% of total calories should be covered by fat, (e) no eating after 7 P.M. or three hours before sleep, and (f) four meals a day. Allsubjects were preconditioned using the above outlined guidelines two weeks before the start of the experiment.

## Results

Basic statistics of the baseline and experimental period are presented in Table 1.

The supervised NW program significantly decreased SBP, and increased upper and lower-body strength in the experimental group. Furthermore, a positive trend in body mass, WC, HC, DBP, VO_2max_, TC, TG and LDL-C levels was observed.

The control group was defined by an unfavorable augmentation in body mass, TG, and LDL-C, and a decrease in lower-body strength.

Although SBP and DBP diminished in both groups, changes in SBP and DBP in the control group were relatively 1.6 and 3.4 smaller than those in the experimental group, respectively.

## Discussion

We studied postmenopausal women who did not take any anti-hypertensive drugs during the 1^st^ and 2^nd^ stage of hypertension (2004), and assessed changes in anthropometric, cardiorespiratory, and functional fitness variables as a function of an 8-week supervised NW program.

A dearth of information on modulation of isolated hypertension (ISH) through physical exercises, leads to inconsistent conclusions; for example, Ferrier et al. (2001) showed that ISH could not be modulated by means of short-term aerobic training, whereas Westhoff et al. (2007) indicated that blood pressure, including ISH, could be modulated through physical exercises.

However, a comparison of current results with previous findings regarding the influence of NW on HRQoL (Hagner et al., 2009) in healthy postmenopausal women, unfolds many positive interrelations between aerobic exercises such as Nordic-walking and parameters defining HRQoL: (a) a non-significant increase in VO_2max_ equal to 2.63 L% in this study versus a significant increase of 4 L% in the latter, i.e., 12-week aerobic NW training which comprised of three 90 min sessions, (b) a non significant decrease of 1.77 L% vs. a significant decrease of 6.90 L% in WC, (c) a non-significant decrease of 4.58 L% vs. a significant decrease of 6.12 L% in TC, (d) a non-significant decrease of 9.69 L% vs. a significant decrease of 3.17 L% in TG, (e) lack of changes in HDL-C vs. a non-significant increase of 6.57 L% in HDL-C, (f) a non-significant decrease of 5.44 L% vs. a significant decrease of 7.38 L% in LDL-C, and (g) a non-significant increase in the BMI of 0.31 L% vs. a significant decrease of 4.77 L% in the BMI.

Although, we were not able to confirm a significant decrease in the BMI of postmenopausal women who had undergone a supervised NW program, as shown in previous studies (Hagner et al., 2009), we observed a beneficial role of a supervised Nordic-walking training program in preventing cardiovascular diseases, caused by ISH.

Additionally, we also showed that the use of walking poles during NW exercises lead not only to an enhancement in oxygen uptake (Hansen and Smith, 2009), but also improves strength of the lower and upper-body.

A conjunction of delineated relations between changes in physiological variables and the NW program with the results of the study on physical fitness and coronary risk factors (Takamiya et al., 2000) makes it transparent that through changes in VO_2max_ SBP can be modulated. We conjectured that NW was superior than W and jogging (Schiffer et al., 2006) for rehabilitation of hypertensive subjects.

## Conclusions

Hypertension has become one of the most profound health problems among the elderly in many societies. This spurred a search for a means, other than pharmaceutical, allowing to maintain proper blood pressure in elderly subjects.

In this study we showed that a supervised 8-week NW program could be effectively employed for averting detrimental changes in SBP in systolic-hypertensive postmenopausal women.

## Figures and Tables

**Table 1:** Differences in anthropometry, serum lipids, physical performance, and functional fitness at baseline and after an 8-week Nordic-walking training program in systolic hypertensive postmenopausal women

**Variable**	Cont (0)	Ex (0)	Cont (8)	p	Ex(8)	L% Con t	L% Ex	p Ex
*Anthropometry*								

**Body weight (kg)**	76.8 (69.6, 80.8)	77.2 (69.6, 79)	78(71, 78.6)		75.4(69, 78.8)	0.67	−1.02	
**BMI (kg/m^2^)**	28.2 (25.2, 32)	28.2 (26, 32.4)	27.4 (26, 32)		28.4 (26, 31.4)	−1.25	0.31	
**WC (cm)**	93.8 (86.6, 97.6)	95 (87, 99)	94.8 (87, 101.4)		91.2(82.6, 96)	0.46	−1.77	
**HC (cm)**	105.8 (103, 113)	107 (103, 114)	108.4(104.8, 119.4)		105(102, 118)	1.05	−0.82	
**WHR**	0.8 (0.8, 0.8)	0.8(0.8, 0.8)	0.8(0.8, 0.8)		0.8(0.8, 0.8)	0	0	
*Cardiovascular fitness*								

**SBP (mmHg)**	149.4 (147.2, 149.6)	145.8(143, 150.4)	142.8(141.2, 150.2)		135.6(126.6, 141.6)	−1.96	−3.15	*a*
**DBP (mmHg)**	86.8(85.2, 87.4)	85.2(83.4, 87.4)	86.2(83, 89)		83.2 (78.4, 88)	−0.3	−1.03	
*Physical fitness*								

**VO_2max_ (ml/kg/min)**	29.6 (24.2, 29.8)	28.8(25.4, 30.4)	29.4(24.2, 31.4)		30.6(28.6, 31.6)	−0.29	2.63	
**Curl (rep/min)**	18 (18, 19)	17.6 (17, 19)	20 (18, 22)		22.6(20.6, 24.6)	4.58	10.86	*a*
**Cst (rep/min)**	16 (15, 16)	15 (15, 16)	15 (15, 17)	*b*	19 (17, 20)	−2.8	10.27	*a*
*Serum lipids*								

**TC (mmol/L)**	6.2 (4.6, 6.4)	6 (4.6, 6.6)	6.2 (4.4, 6.4)		5.4 (4.4, 6)	0	−4.58	
**TG (mmol/L)**	1 (0.8, 1.6)	1 (0.8, 1.4)	1.2 (1, 1.4)	*b*	0.8 (0.8, 1)	7.92	−9.69	
**HDL (mmol/L)**	1.8 (1.8, 2)	2 (1.6, 2)	2 (1.6, 2)		2 (1.4, 2.2)	4.58	0	
**LDL (mmol/L)**	3.2 (2.8, 4)	3.4 (2.6, 4)	3.6 (2.6, 4)		3 (2.6, 3.4)	5.12	−5.44	

BMI = Body Mass Index; Ex = exercise group; Cont = control group; TC = total cholesterol; TG = triglycerides; HDL-C = high-density cholesterol; LDL-C = low-density cholesterol; WC = waist circumference; HC = hip circumference; WHR = waist/hip ratio; SBP = systolic blood pressure, DBP = diastolic blood pressure; VO_2max_ = maximal oxygen capacity; Curl = arm bend test, Cst =chair stand test;

a – p-value less than 0.05 for differences in medians induced by the experiment;

b - p-value less than 0.05 for differences in means/medians between the experimental group and control group for the specific period of the experiment.
